# Equity in maternal and child health care utilization in Guangdong province of China 2009–2019: A retrospective analysis

**DOI:** 10.3389/fpubh.2022.963344

**Published:** 2022-09-13

**Authors:** Xin Wang, Yingxian Zhu, Jia Liu, Yuanzhu Ma, Stephen Birch

**Affiliations:** ^1^School of Public Health, Sun Yat-sen University, Guangzhou, China; ^2^Guangdong Women and Children Hospital, Guangzhou, China; ^3^Centre for the Business and Economics of Health, University of Queensland, Brisbane, QLD, Australia

**Keywords:** maternal and child health, service utilization, equity, Gini coefficient, Theil index, China

## Abstract

**Background:**

Equity is the principal challenge of maternal and child health care (MCH) across the world, especially in China. Existing researches focused on equity in MCH resources and outcomes. There is an evidence gap regarding equity of MCH services utilization, revealing the black box between equity in MCH resources and MCH outcomes. In the study, we evaluate the changes of equity in integrated MCH service utilization in Guangdong province of China during 2009–2019.

**Methods:**

Data used in this study are from the Guangdong Maternal and Child Health Routine Reporting System and the Guangdong Health Statistical Yearbook (2009–2019). The Gini coefficient (G) and Theil index (T) were employed to assess equity and source of inequity in four geographic regions of Guangdong province.

**Results:**

Generally, among the integrated MCH care, coverage of pre-pregnancy care (<50%) is lower than in other stages. In the past decade, inequity of MCH care in the Equalization of Essential Public Health Service (EEPHS) program has gradually reduced to *G* < 0.1. Screening of genetic metabolic disease and of hearing showed largest reductions of inequity (*G* reducing from 0.3–0.4 to 0.03–0.04). Inequity in reproductive health tests for brides-to-be, psychological assessment and consultation, education classes for mother-to-be and health management of children under 3 were mainly contributed by intra-region disparities in 2019.

**Conclusion:**

Equity has gradually improved in the last decade in Guangdong. The national EEPHS program and the Integrated Prevention of Mother-to-Child Transmission of HIV, Syphilis and HBV of Guangdong have played important roles in reducing inequity in MCH service utilization. Further strategies, targeting pre-pregnancy reproductive healthcare, psychological assessments and consultations for the pregnant and education classes for mothers-to-be, should be taken to promote coverage and equity.

## Introduction

Maternal and child health (MCH) care is an essential component to achieve the third and tenth Sustainable Development Goals, promoting wellbeing for all at all ages and reducing inequalities (1). The last two decades witnessed a significant improvement in MCH worldwide during the implementation of Millennium Development Goals (MDG) (2). However, the maternal mortality ratio, the proportion of mothers that do not survive childbirth, in developing regions is still 14 times higher than in the developed regions (3). Meanwhile, children in the poorest 20% of the populations are still up to three times more likely to die before their fifth birthday than children in the richest quintile over the world (4). Therefore, promoting equity of MCH care utilization and MCH outcomes has been the main driver to improve sustainable MCH improvement (5).

There has been a remarkable improvement in MCH in China over recent decades. Maternal deaths declined by 79.4%, from 111.0 per 100,000 live births in 1990 to 18.3 per 100,000 live births in 2018. And the mortality rate among infants and children under-5 declined by 88.2% (50.2‰ in 1991, 6.1‰ in 2018) and 86.2% (61.0‰ in 1991, 8.4‰ in 2018) respectively during the same period (6). Despite the progress in MCH outcome in China, substantial disparities remain. The mortality rate for children under-5 in rural regions and urban regions were 10.2 and 4.4‰ in 2018, and the disparity also remains in eastern, central and western China (4.2, 7.2, and 12.7‰) (6). Inequity in MCH care utilization explained partial inequity in MCH outcome among different regions in China. Reported by a national research with 31 provinces/municipality in China, the coverage of health management of the pregnant women in Beijing was 96.0% in 2018, while the coverage in Tibet was 44.2% (7).

Several strategies have been adopted for reducing inequity in MCH care utilization since the new round of comprehensive health reform 2009 (8). Firstly, integrating MCH services and interventions have been incorporated into the Equalization of Essential Public Health Service (EEPHS) program nationwide. In the essential public health service package, MCH care consists of free physical examination before pregnancy to promote healthy birth and child development for all rural couples, systematic health management for pregnant women and children, subsidies for hospitalized delivery to rural women, free treatment for HIV-infected pregnant women, neonatal screening, free planned child immunization, and a child nutrition improvement program for children in poor areas (9). Secondly, the EEPHS package is provided free for residents, co-funded by the central and local governments. That is, unsustainable special MCH fund before 2009 was replaced by sustainably fund for the EEPHS program (10). Moreover, $1.3 billion was invested by the central government for development of MCH hospitals nationwide 2016–2018. Thirdly, local governments were encouraged to subsidize other MCH services in addition to for those in the EEPHS package based on local financial capacity and health needs. A research in 2020 showed that, despite the small reductions in inequity of MCH outcomes nationwide in China, inequity is increasing in certain regions (11).

Several researches have analyzed equity related to MCH at the national, provincial or city level in China (7, 9, 11). Regarding location of the researches, most of them focused on the relatively underdeveloped western and central China, omitting the eastern provinces with a large gap between rich and poor (9). Regarding analytical indicators, most researches used Gini coefficients, concentration indices (CI) and concentration curves (CC), Theil indices and health resource density index (12). Regarding the subjects of inequity, most studies focused on inequity in MCH health resources and health outcomes. Several researches measured inequity in maternal care or newborn care, such as antenatal care, hospital delivery and breastfeeding (13, 14). However, there is strong international consensus on the need for strengthening integrated MCH care. Defined by Partnership for Maternal, Newborn and Child Health (PMNC), integrated MCH care encompasses a continuum of essential interventions that should be accessible to mothers, newborns and children at household, community, district and national levels, as well as continuum that follows through the life-cycle of maternal, newborn and child health (15). Interventions across integrated MCH care were framed based on available data in existing researches. To track progress toward universal coverage for reproductive, maternal, newborn and child health, Countdown to 2030 Collaboration covered 18 interventions (grouped into seven dimensions, pre-pregnancy, pregnancy, birth, postnatal, infancy, childhood and environment) based on data available in the 81 Countdown countries (5). Kerber et al. proposed that integrated care for maternal, newborn and child health were supposed to connect care across before pregnancy, pregnancy, birth, postnatal for mother and newborn, infancy and childhood (16). Few research framed interventions of integrated MCH care or analyzed inequity in MCH service utilization from the perspective of integrated care in China. Therefore, there is an evidence gap regarding equity in integrated MCH services utilization in China.

**Figure 1:**
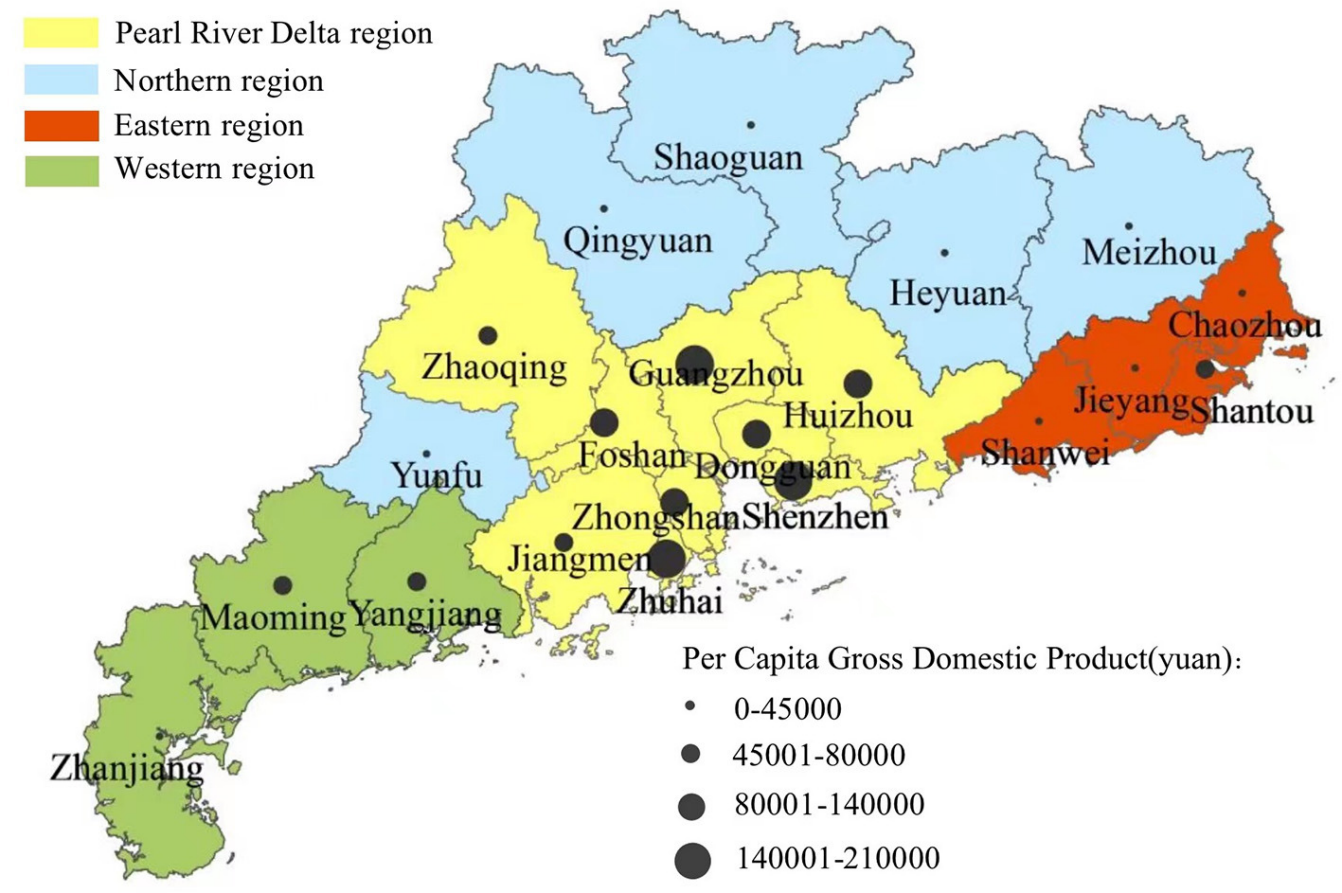
Map of four regions in Guangdong province.

Guangdong province is located in the southeast China, with four regions geographically (Figure 1), the Pearl River Delta region, the northern region, the eastern region and the western region. It has a gross domestic product (GDP) ranked 1^st^ among 31 mainland provinces and municipalities for the last 30 years. The economy supports more investment into MCH than other provinces, providing more free integrated care for pregnant women and newborn children. However, per ca-pita disposable income in the northern region (¥21,288) was less than half of that in the Pearl River Delta region (¥47,911) in 2018 (17). Imbalances in economic development affects equity of resource distribution and care utilization of MCH care within the province. Equity of MCH services utilization among the four regions, is a significant challenge to continuously improve MCH in Guangdong. What's the equity in integrated MCH service utilization of Guangdong province nowadays? And whether the changing trend of the inequity in the last 10 years could provide evidence for the sustainable development of MCH in the future. This study aims to show the inequity in integrated MCH service utilization of Guangdong province in 2019, analyze changes of the inequity and its sources among different regions during 2009–2019, and provides evidence for MCH development in China and other low- and middle- income countries.

## Materials and methods

### Setting

The Health Commission of Guangdong has prioritized the integration of MCH care delivery in recent years (18). Under the EEPHS program, some MCH care are provided free for residents in Guangdong, including antenatal care (at least five visits), health management of the pregnant women, postnatal visits for babies and mothers, health management of children under 3 and health management of children under 7. Additionally, in response to the national Integrated Prevention of Mother-to-Child Transmission of HIV, Syphilis and HBV program (iPMTCT), the number of counties or districts providing free iPMTCT care increased from 10 in 2009 to all counties/districts in 2015. The iPMTCT program was fully funded by the central governments, providing free care for all pregnant women. Although provided free, the reproductive health tests for brides-to-be is not mandatory for women to attend. Partially funded by Guangdong government, screening of genetic metabolic disease and hearing has been provided for newborn babies with an out-of-pocket cost of 20% since 2015. Increasingly more attention has been paid to the importance of psychological assessments and consultations and education classes for mother-to-be, but this hasn't been accompanied by financial support, care guidelines or management of care delivery in the health care systems.

### Data source and indicators

**Figure 2:**
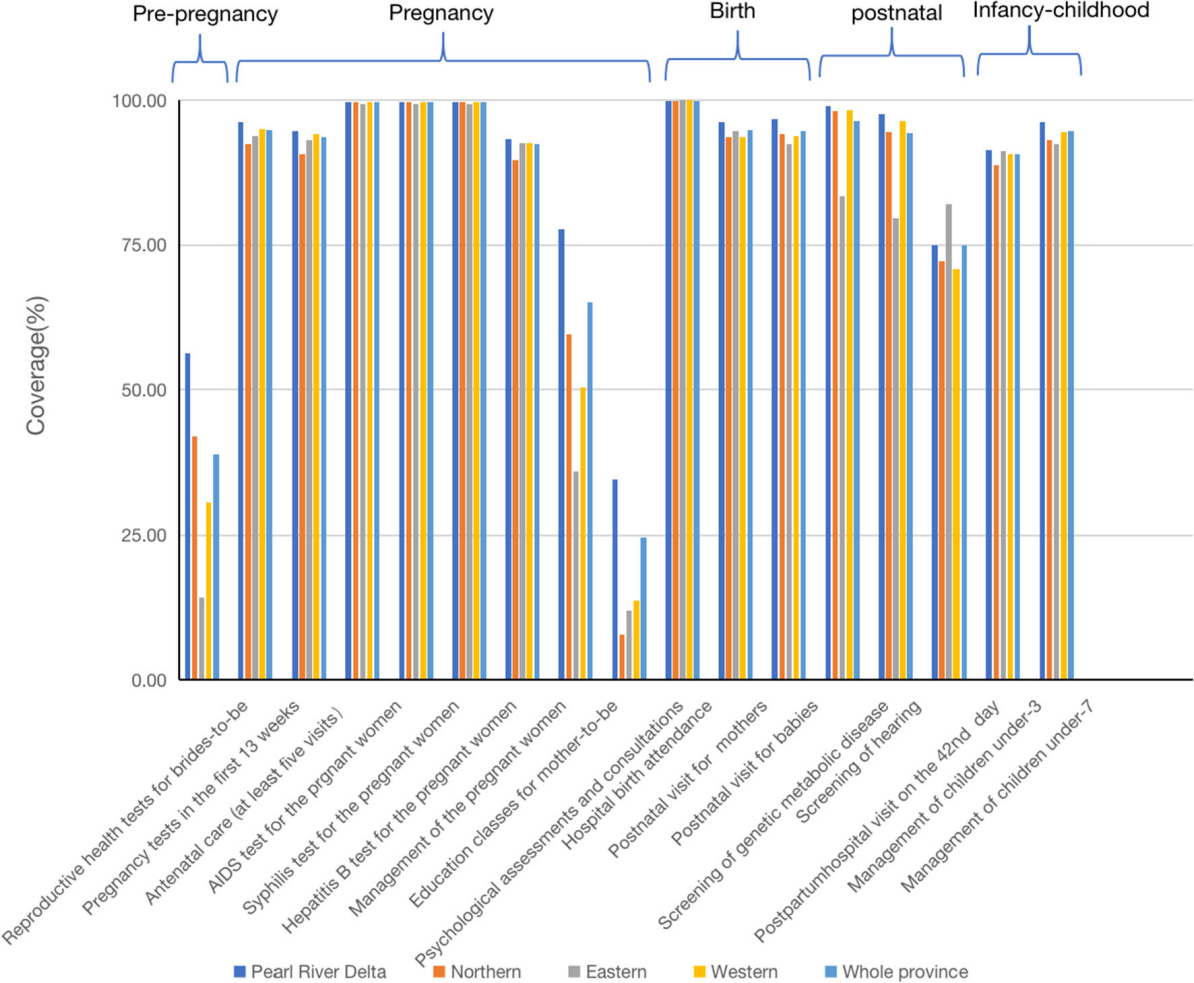
Coverage of integrated MCH care, from pre-pregnancy to childhood, in four regions and the whole Guangdong 2019.

Protocol for this study has been approved by Ethics Committee of School of Public Health, SUN Yat-Sen University. Data of this study came from the Guangdong Maternal and Child Health Services Surveillance System (2009–2019). This surveillance system is a facility-based data collection network, focusing on collecting data about health resource and services utilization of MCH. All health facilities in Guangdong providing MCH care are responsible for uploading their own routine data. Data are collected and audited by staff in the health commissions of county/district level, city level and province level, for quality control. All of the 17 indicators regarding MCH care utilization (acrossing pre-pregnancy, pregnancy, birth, postnatal, infancy-childhood) were used to describe integrated MCH care utilization in 2019 (Figure 2). Ten of the 17 indicators, with coverage lower than 99%, were adopted for evaluating changes in equity in MCH care utilization among different regions in Guangdong province during 2009–2019. The ten indicators consist of five prenatal care (productive health test among brides-to-be, antenatal care (at least five visits), health management of the pregnant women, psychological assessments and consultations for the maternal, education classes for mother-to-be), two perinatal care (postnatal visits for the babies and the mothers), and three postnatal care (screening of genetic metabolic disease for the babies, screening of hearing for the babies, health management of children under 3). We chose 2009 as the baseline for our analyses because the new round of health reforms, including the EEPHS program, started in 2009. However, in the cases of antenatal care (at least five visits) and psychological assessments and consultations for pregnant women and education classes for mother-to-be data were only available for the period commencing 2011 and 2016 respectively. Additionally, we collected number of newborns in each facility from the Guangdong Maternal and Child Health Services Surveillance System.

### Data analysis

We started by describing integrated MCH care utilization at the provincial level in 2019, to show the status and performance of MCH care development. Then, Gini coefficients and Theil indices were used to evaluate changes of equity in MCH care utilization among four regions in Guangdong, and to explore sources of the inequity in partial MCH care utilization in some regions during 2009–2019.

### Gini coefficient

Derived from the Lorenz Curve, the Gini coefficient is the ratio of the area between Lorenz Curve and the 45° line to the whole area below the 45° line (19). The formula to calculate Gini coefficient is presented as follows:


(1) $$\begin{array}{c} G=1-\sum_{i=0}^{k=1}\left(Y_{i+1}+Y_{i})(X_{i+1}-X_{i}\right) \end{array}$$

*Y*_*i*_ is the cumulative percentage of MCH care utilization (each of the ten indicators used to assess the equity) in the *ith* region. *X*_*i*_ is the cumulative percentage of newborns in the *ith* region. *k* is the total number of regions (20). *G* is the value of Gini coefficient.

The value of Gini coefficient ranges from 0 to 1, with higher score indicating greater inequity. Generally, a Gini coefficient < 0.3 indicates a high level of equity, 0.3–0.4 represents acceptable equity, 0.4–0.6 refers to an alert of inequity, and >0.6 indicates a several inequity (21).

### Theil index

The Theil index is regarded as a superior to the Gini for analyzing the source of inequity (22). It decomposes inequity into intra- and inter-region inequity. The Theil index is a relative indicator without available assessment standards. However, in general, smaller *T* values indicate greater equity.

The Theil index, *T*, is given by:


(2) $$\begin{array}{c} T=\sum_{i=1}^{n}P_{i}\ln\frac{P_{i}}{Y_{i}} \end{array}$$

*P*_*i*_ is the proportion of the *ith* city's newborns accounting for the total newborns in the region. *Y*_*i*_ is the proportion of the region's MCH care utilization in each city.

Additionally, *T* can be divided into *T*_*inter*_ and *T*_*intra*_ as follows:


(3) $$\begin{array}{r} T=T_{inter}+T_{intra} \end{array}$$

(4) $$\begin{array}{c} T_{intra}=\sum_{g=1}^{k}P_{g}T_{g} \end{array}$$

(5) $$\begin{array}{c} T_{inter}=\sum_{g=1}^{k}P_{g}\;\ln\frac{P_{g}}{Y_{g}} \end{array}$$

*T*_*g*_ is the T value of the *gth* region. *P*_*g*_ is the proportion of the province's number of newborns contributed by each region. *Y*_*g*_ is the proportion of the province's MCH care utilized in each region. The contribution of intra- and inter-region inequity can be calculated by *T*_*intra*_/*T* and *T*_*inter*_/*T*.

All the analyses were performed using STATA 15.

### Patient and public involvement

At the outset of the study, the research team engaged eight health providers and six staff working on data management in Guangdong Women and Children Hospital, to consultant for advise on study design, process, and outcomes of interest.

## Results

### Integrated MCH care coverage in Guangdong 2019

Figure 2 shows coverage of integrated MCH care, from pre-pregnancy to childhood, in four regions and the whole Guangdong 2019. Generally, comprehensive MCH care was provided and strictly monitored in Guangdong. Across the range of MCH care, coverage of care before pregnancy is lower than care in other stages of MCH care. AIDs testing, syphilis testing and Hepatitis B testing during pregnancy, and hospital birth attendance share the highest coverage rates (over 99%) in Guangdong province and its four regions. Another five types of care covered over 90% of the newborns, pregnancy tests in the first 13 weeks, antenatal care (at least five visits), postnatal visits for mothers, postnatal visits for babies, and health management of children under 7. Differences among the four regions for care with low coverage levels are larger than that for care with coverage rates over 90% (see Figure 2).

### The equity of MCH care utilization in four regions 2009–2019

**Figure 3:**
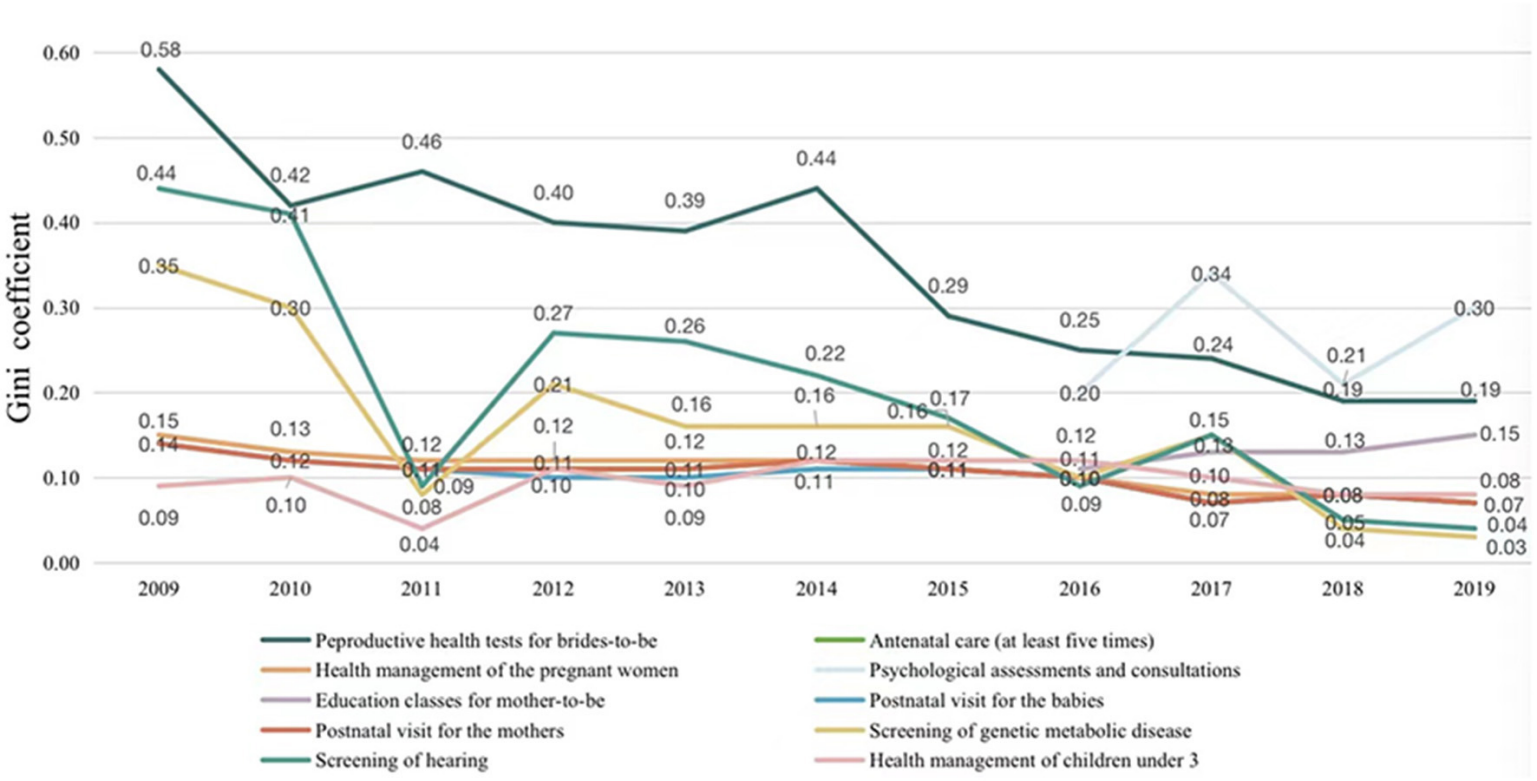
Gini coefficient of MCH care utilization by population 2009–2019.

Figure 3 represents the trends of inequity in the use of ten MCH services from 2009 to 2019 (antenatal care with at least five visits: 2011–2019; psychological assessments and consultations: 2016–2019; education class for mother-to-be: 2016–2019). Inequity of MCH services in the EEPHS program, antenatal care with at least five visits, health management of the pregnant women, postnatal visit for mothers, postnatal visit for babies and health management of children under 3, have gradually reduced to *G* < 0.1 over the 11 years studied. Screening of genetic metabolic disease and screening of hearing showed similar and big reduction of inequity between 2009–2019 and indicated only minimal levels of inequity remained in 2019. In spite of gradual reductions in inequity, reproductive health tests for brides-to-be indicated the second largest level of inequity in 2019. Psychological assessments and consultations showed the largest inequity in use among all MCH care, without any evidence of significant reduction.

### The sources of inequity in four MCH care utilization 2009–2019

**Figure 4:**
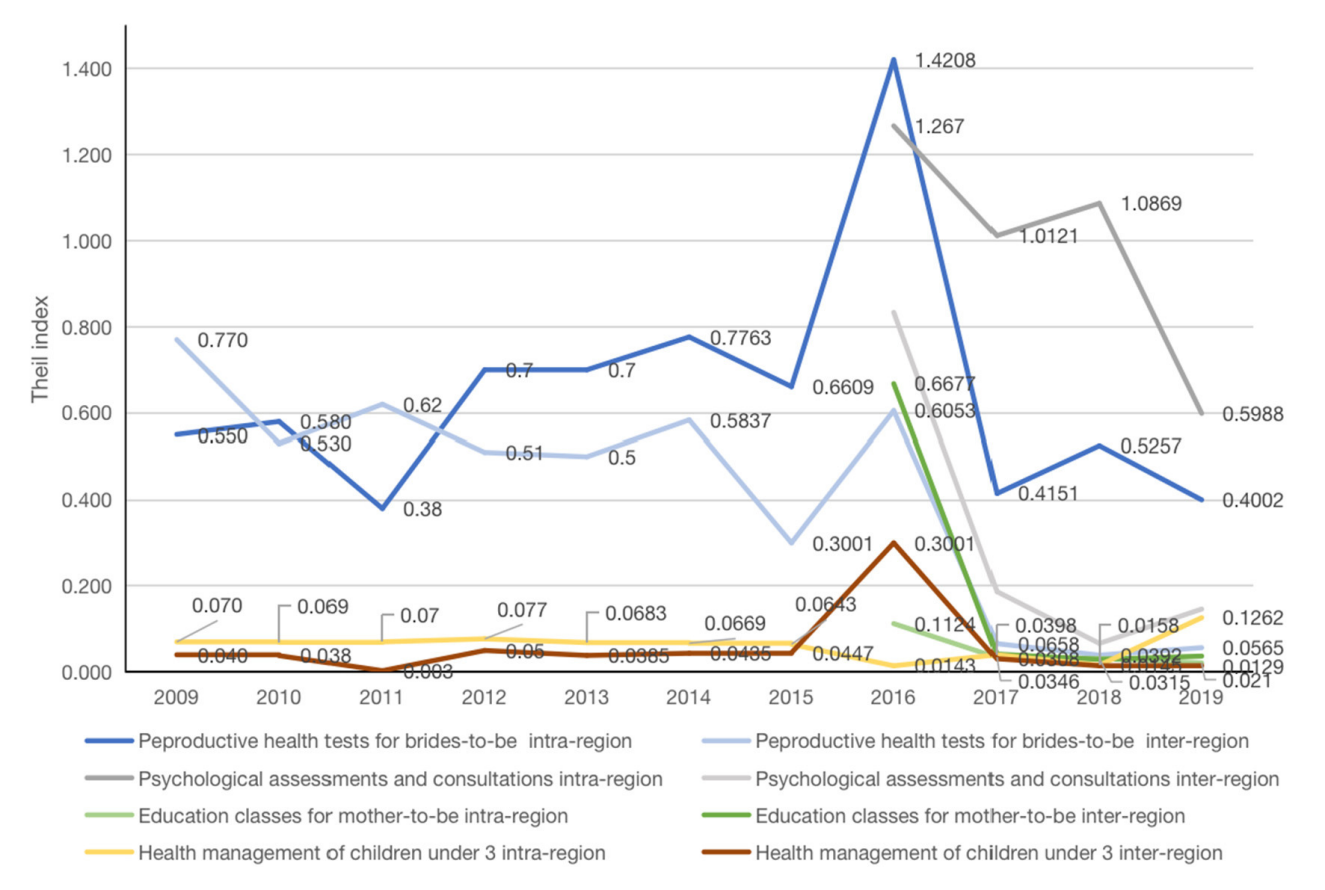
The sources of inequity in four MCH care utilization 2009–2019.

The inequity, sources of inequity and their contribution for the ten MCH care utilization in Guangdong during 2009–2019 are presented in Supplementary Tables 1–3. Figure 4 shows the trends of shares attributed to inter- and intra-region disparities from 2009 to 2019 in the four MCH services, with the highest Gini coefficients in 2019. It indicates that, in addition to education classes for mother-to-be, inequity in three other services are mainly from intra-region disparities. Further analysis is needed to reveal which regions the inequity arises. Although reproductive health tests among brides-to-be has been provided and monitored since 2009, the shares attributable to the intra- and inter-region disparities remained high until before falling steadily thereafter. The shares attributable to the inter- and intra- region disparities of psychological assessments and consultations have gradually reduced since 2016, but more attention should be paid to intra-region disparity in this service (*T*_*intra*_ = 0.60).

### The decomposition of intra-region differences in four MCH care utilization 2009–2019

**Figure 5:**
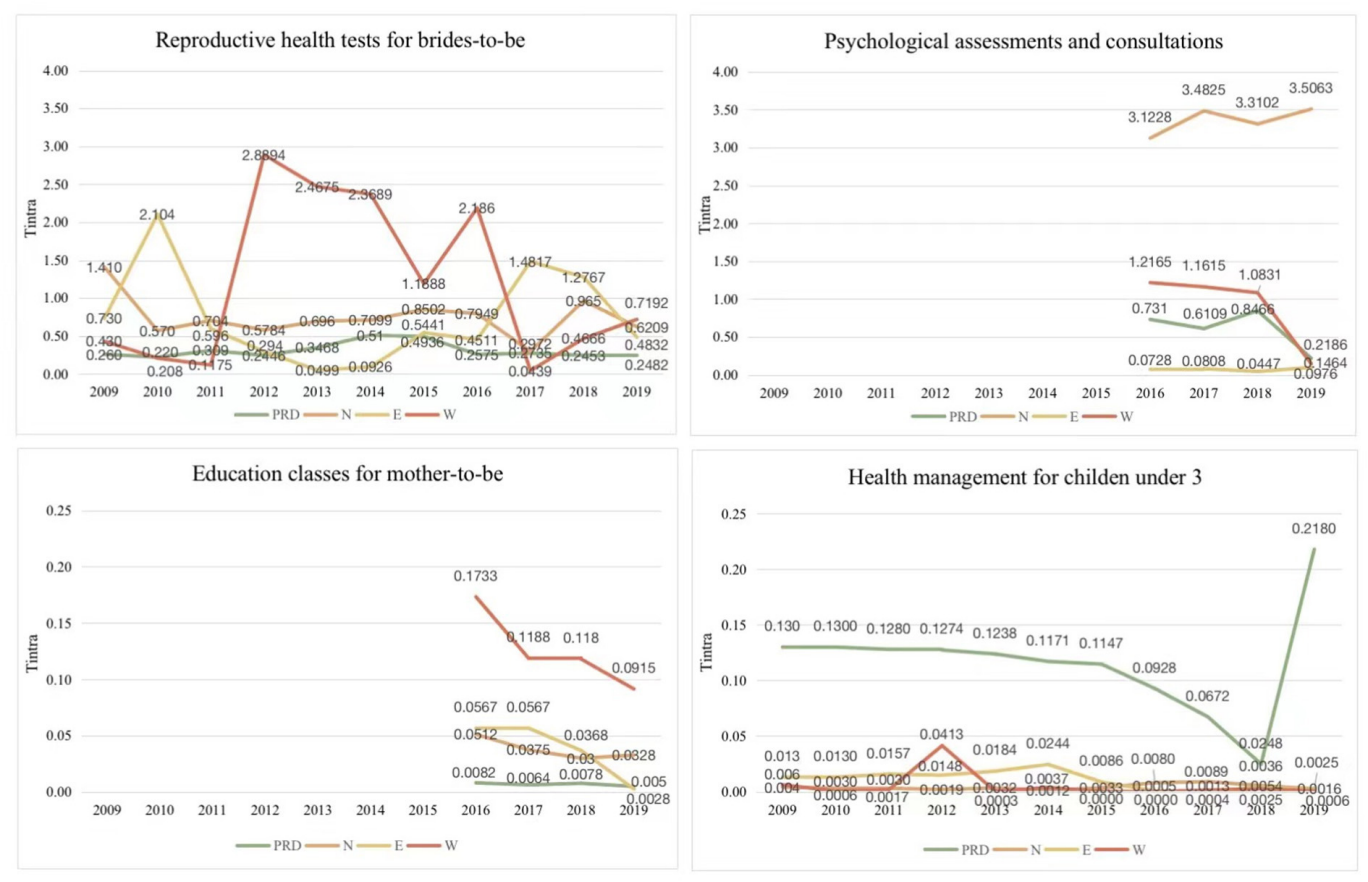
The decomposition of intra-region differences in four MCH care utilization 2009–2019. PRD, Pearl River Delta region; N, northern region; E, eastern region; W, western region.

Based on the results shown in Figure 4, intra-region disparities were decomposed in the four MCH services (Figure 5). Over the last decade, in regard to reproductive health tests among brides-to-be, the shares attributed to disparities among the four regions showed the similar change with no obvious trend. In 2019, differences in the intra-western region had the largest contribution (*T* = 0.72) to inequity of reproductive health tests among brides-to-be, followed by the northern region (*T* = 0.62). Between 2016 and 2018, intra-region differences of the four regions in psychological assessments and consultations were relatively constant, but differed significantly across regions with intra-northern region shared the largest contribution (*T*≈3.20), followed by intra-western region (*T*≈1.00), intra-Pearl River Delta region (*T*≈0.70)and intra-eastern region (*T*≈0.07). In 2019, sharp reductions in shares of intra-region differences were observed for psychological assessments and consultations in the western region and the Pearl River Delta, which contrasted with slight increases in the northern and eastern regions. For education classes for mother-to-be, the shares attributed to intra-western region ranked the highest among the four regions during 2016–2019. In 2009–2019, the shares attributed to intra-the Pearl River Delta region in health management for children under-3 were much higher than those intra- other three regions, and with a jump in 2019.

## Discussion

### Principal findings

Current areas of concern regarding equity of integrated MCH care utilization in Guangdong province are pre-pregnancy reproductive health tests, psychological assessments and consultations and education classes for mother-to-be. It has been demonstrated that the continuum of care for MCH that recognizes a tight inter-relationship between maternal, newborn and child health at different time periods and locations is key toward reducing inequity in health (23). Therefore, it's necessary to monitor and address gaps in integrated care and inequity in the distribution of MCH care in Guangdong and the four regions.

The results show that significant progress has been made in terms of equity of MCH care utilization under the EEPHS program during the study period in Guangdong. Generally, the Gini coefficients of the five MCH services included in the EEPHS service package have been gradually reducing to below 0.10. This finding is consistent with results of Gao and colleagues who concluded that EEPHS program reduced inequity of MCH care in the western region of China in 2017 (24). Jiang also revealed EEPHS's role in promoting geographical equity in Chongqing (25). In a review of China's MCH care development, Guo concluded that the EEPHS program had played a key role in the protection of women's and children's health and promotion of health equality (10). As the main provider in the EEPHS program, primary health institutions play an important role in reducing inequity in MCH care utilization (26). Research in Ethiopia also indicated that expansion of PHC facilities might have an important role in narrowing the geographic and demographic gaps in health service utilization for selected MCH interventions (27). In addition to meeting requirements of the national EEPHS program, the Health Commission of Guangdong province implemented additional strategies (28). Firstly, paying close attention to MCH care provision in rural areas for the poor and migrant populations. Secondly, strengthening the capacity of MCH care provision in primary health networks (consisting of county/district hospitals, township health centers/community health centers, village clinic/community health stations) by introducing appropriate MCH care technologies. Thirdly, establishing a strict hierarchical performance assessment system.

AIDs, Syphilis and Hepatitis B testing has covered over 90% of the pregnant women since 2014, and the coverage rate in 2019 was over 99% each of the four regions of Guangdong province indicating close to full equity. This achievement demonstrates that special project with specific sustainable funding, independent management and assessment, and specific guidelines provide a promising strategy for reducing infectious diseases affecting MCH in a short time period. It has also been demonstrated by implementation research funded by international agencies in low-income countries (29). The iPMTCT program was established in 2010, to control mother-to-child transmission. Subsequently, a specific work plan for the program was issued, to ensure national and local funding, qualified professional technical personnel and standard working guidelines. Some physicians have reported higher efficiency and better effectiveness of specific programs compared to more opportunistic practice and service delivery (30).

To further promote MCH care and reduce inequity in Guangdong province, it is necessary to tackle inequity from intra-region differences in three MCH services (reproductive tests for the brides-to-be, psychological assessments and consultations and health management of children under 3) and inequity from inter-region differences in one MCH service (education classes for mother-to-be). Some reasons for inequity were revealed by reviewing related health policies and researches. Policies about reproductive tests for the brides-to-be has ranged from compulsory testing with user payment, voluntary testing with user payment and free voluntary testing in Guangdong during the last two decades, which might lead to instability and inequity of this care in different time periods and different regions. As an emerging public health problem, mental health of the pregnant women and new mothers was not paid adequate attention in Guangdong or nationwide. Some indicators related to mental health of perinatal women have been adopted in construction of guidelines for MCH institutions since 2015 (31, 32). It was illustrated that the large migrant population (over 26.48 million in 2016) in the Peal River Delta region resulted in relatively low coverage of health management for children under 3 and relative larger contributions to inequity than in the other three regions (33).

### Policy implications

Based on the identification of the underlying sources of inequity in different MCH services, measures should be taken to further reduce geographica inequity and promote health of the pregnant women, new mothers and children in Guangdong province. Firstly, in regard to reproductive tests for brides-to-be, improving health literacy of the public, strengthening publicity by social media, and facilitating access to the test will benefit care coverage and reduce inequity (34). Secondly, with respect to psychological assessments and consultations for the new mothers, integrating the tests into the service package of the EEPHS program and improving psychological skills of staff in primary health care institutions would offer potential improvements. The standardized implementation of the EEPHS program in the past decade provides an opportunity to support these improvements. Thirdly, to enhance health education for pregnant women, Guangdong province has used “number and proportion of pregnant women who attends education classes for mother-to-be” as a monitoring indicator since 2016. Digital solutions, such as online maternity school by applications, as well as telephone and We-Media modes of communication, could continue to improve the capacities and skills of pregnant women and new mothers in the COVID-19 period (35). Lastly, it appears important to implement cross-city health management and information sharing for children under 3 in the whole province for migrant children. With a booklet of vaccination information, a child could get appropriate vaccination in every qualified institution across the nation. This mode might provide reference for health management for migrant children under 3.

The experience of promoting equity in MCH care in Guangdong also has some implications for other provinces of China and other low-and middle-income countries. Firstly, promoting universal MCH coverage is the precondition for improving equity of MCH care. Vulnerable new mothers and children, such as those living in rural area, in families with poor financial situation, or migrant laborers, deserves more attention. Secondly, providing regular time-driven MCH care, such as health management and prenatal examinations are required through primary care settings. Adequate qualified medical staff in primary care institutions will ensure quality of regular MCH care, improve coverage and reduce inequity in care. Finally, special projects with specific sustainable funding, independent management and assessment was the most promising strategy for promoting coverage and reducing inequity in urgent MCH problems.

### Strengths and limitations

This study not only analyzed the changes of equity in MCH service utilization but also explored sources of the inequity in a province of China, using population-based longitudinal data from 2009 to 2019. Comparison of inequity among four regions and different services, visualization of the changing trends, and decomposition of the inter- and intra- inequity provided evidence for policy making in regions and cities. Moreover, comparing with existing researches, it has two strengths. On one hand, it's the first time to define integrated MCH services in Chinese healthcare systems. On the other hand, it represents the first study analyzing inequity of psychological assessments and consultations and education classes for mother-to-be, as these two services were not included in the surveillance systems by any other province or municipality of mainland China. However, there are some limitations in the study. First, indicators in this study are based on the Guangdong Maternal and Child Health Services Surveillance System. Some MCH services, excluded by the system, could also represent utilization of integrated MCH care. Further work is underway to address interconnection of information systems in different MCH institutions (36). Second, this study pooled the urban and rural cases in the same city and region, could not provide evidence for improving inequity by reducing urban and rural disparities. Third, this study analyzed inequity in MCH service utilization and the changes from the perspective of geography. However, it provided limited reasons for the inequity. Further analyses of reasons for the inequity, such as related MCH services delivery in different regions at different time period, are needed to be conducted.

## Conclusion

Our results show that coverage of most MCH care in Guangdong province is higher than average levels of the whole country. It's necessary to promote coverage and equity of the three types of care, pre-pregnancy reproductive health tests, psychological assessments and consultations for pregnant women and education classes for mother-to-be. Further strategies, targeting reasons for under-utilization of the three types of care, should be taken to fill the knowledge gap. In addition to the above three services, equity in other MCH services in EEPHS has gradually improved in the last decade. The national EEPHS program and the specific projects of AIDs, Syphilis and Hepatitis B testing of Guangdong has played an important role in reducing inequity and improving health of new mothers and children, providing references for other low- and middle-income countries facing similar challenges.

## Data availability statement

The datasets used and analyzed during the current study are available from the corresponding author on reasonable request.

## Ethics statement

The protocol for the research project has been approved by Ethics Committee of School of Public Health, SUN Yat-sen University (ref 2020 No. 073). Written informed consent was obtained from the individual(s) for the publication of any potentially identifiable images or data included in this article.

## Author contributions

SB and YM: conceptualization. XW: funding acquisition and writing—original draft. YZ and YM: data collection. JL and XW: data analysis. YM, YZ, JL, and SB: writing—review and editing. All authors contributed to the article and approved the submitted version.

## Funding

This work was supported by the China Medical Board (Grant Number 21-437). The funder had no role in preparation of the manuscript or decision to publish.

## Conflict of interest

The authors declare that the research was conducted in the absence of any commercial or financial relationships that could be construed as a potential conflict of interest.

## Publisher's note

All claims expressed in this article are solely those of the authors and do not necessarily represent those of their affiliated organizations, or those of the publisher, the editors and the reviewers. Any product that may be evaluated in this article, or claim that may be made by its manufacturer, is not guaranteed or endorsed by the publisher.

## Author disclaimer

We certify that the materials reported in this paper is not under consideration for publication elsewhere and its publication is approved by all authors and responsible authorities where the work was carried out.
