# Supplementary material for: Equity in maternal and child health care utilization in Guangdong province of China 2009–2019: A retrospective analysis
Source: Front Public Health. 2022 Sep 13;10:963344. doi: 10.3389/fpubh.2022.963344 (PMC9513387; doi:10.3389/fpubh.2022.963344)
Supplement: Supplementary file 1 [file Data_Sheet_1.docx]

**Supplemental Table 1 Sources of inequity of MCH care utilization 2009-2019**

|  | **Sources of inequity** | **2009** | **2010** | **2011** | **2012** | **2013** | **2014** | **2015** | **2016** | **2017** | **2018** | **2019** |
| --- | --- | --- | --- | --- | --- | --- | --- | --- | --- | --- | --- | --- |
| **Reproductive health tests for brides-to-be** | intra-region | 0.550 | 0.580 | 0.38 | 0.7 | 0.7 | 0.7763 | 0.6609 | 1.4208 | 0.4151 | 0.5257 | 0.4002 |
|  | inter-region | 0.770 | 0.530 | 0.62 | 0.51 | 0.5 | 0.5837 | 0.3001 | 0.6053 | 0.0658 | 0.0392 | 0.0565 |
|  | Contribution of intra-region（%） | 41.67 | 52.25 | 38.00 | 57.85 | 58.33 | 57.08 | 68.77 | 70.12 | 86.32 | 93.06 | 87.63 |
|  | Contribution of inter-region（%） | 58.33 | 47.75 | 62.00 | 42.15 | 41.67 | 42.92 | 31.23 | 29.88 | 13.68 | 6.94 | 12.37 |
| **Antenatal care (at least five times)** | intra-region |  |  | 0.07 | 0.07 | 0.0613 | 0.0539 | 0.051 | 0.0121 | 0.0227 | 0.0112 | 0.009 |
|  | inter-region |  |  | 0.06 | 0.06 | 0.0503 | 0.0553 | 0.0435 | 0.3074 | 0.0235 | 0.0179 | 0.0155 |
|  | Contribution of intra-region（%） |  |  | 53.85 | 53.85 | 54.93 | 49.36 | 53.97 | 3.79 | 49.13 | 38.49 | 36.73 |
|  | Contribution of inter-region（%） |  |  | 46.15 | 46.15 | 45.07 | 50.64 | 46.03 | 96.21 | 50.87 | 61.51 | 63.27 |
| **Health management of the pregnant women** | intra-region | 0.090 | 0.070 | 0.08 | 0.08 | 0.0686 | 0.0603 | 0.0535 | 0.0124 | 0.0251 | 0.0123 | 0.0094 |
|  | inter-region | 0.090 | 0.080 | 0.07 | 0.06 | 0.0563 | 0.0611 | 0.047 | 0.3002 | 0.027 | 0.0197 | 0.0157 |
|  | Contribution of intra-region（%） | 50.00 | 46.67 | 53.33 | 57.14 | 54.92 | 49.67 | 53.23 | 3.97 | 48.18 | 38.44 | 37.45 |
|  | Contribution of inter-region（%） | 50.00 | 53.33 | 46.67 | 42.86 | 45.08 | 50.33 | 46.77 | 96.03 | 51.82 | 61.56 | 62.55 |
| **Psychological assessments and consultations** | intra-region |  |  |  |  |  |  |  | 1.267 | 1.0121 | 1.0869 | 0.5988 |
|  | inter-region |  |  |  |  |  |  |  | 0.8335 | 0.1867 | 0.0665 | 0.1462 |
|  | Contribution of intra-region（%） |  |  |  |  |  |  |  | 60.32 | 84.43 | 94.23 | 80.38 |
|  | Contribution of inter-region（%） |  |  |  |  |  |  |  | 39.68 | 15.57 | 5.77 | 19.62 |
| **Education classes for mother-to-be** | intra-region |  |  |  |  |  |  |  | 0.1124 | 0.0346 | 0.0315 | 0.021 |
|  | inter-region |  |  |  |  |  |  |  | 0.6677 | 0.0415 | 0.0292 | 0.0369 |
|  | Contribution of intra-region（%） |  |  |  |  |  |  |  | 14.41 | 45.47 | 51.89 | 36.27 |
|  | Contribution of inter-region（%） |  |  |  |  |  |  |  | 85.59 | 54.53 | 48.11 | 63.73 |
| **Postnatal visit for the mothers** | intra-region | 0.070 | 0.060 | 0.06 | 0.06 | 0.0561 | 0.0505 | 0.0486 | 0.0115 | 0.0214 | 0.0109 | 0.0082 |
|  | inter-region | 0.060 | 0.060 | 0.05 | 0.05 | 0.0453 | 0.0515 | 0.0405 | 0.3101 | 0.0233 | 0.0187 | 0.0154 |
|  | Contribution of intra-region（%） | 53.85 | 50.00 | 54.55 | 54.55 | 55.33 | 49.51 | 54.55 | 3.58 | 47.87 | 36.82 | 34.75 |
|  | Contribution of inter-region（%） | 46.15 | 50.00 | 45.45 | 45.45 | 44.67 | 50.49 | 45.45 | 96.42 | 52.13 | 63.18 | 65.25 |
| **Postnatal visit for the babies** | intra-region | 0.070 | 0.063 | 0.06 | 0.062 | 0.0537 | 0.0504 | 0.0488 | 0.011 | 0.0207 | 0.0112 | 0.0077 |
|  | inter-region | 0.060 | 0.058 | 0.053 | 0.05 | 0.0447 | 0.0478 | 0.04 | 0.3133 | 0.0232 | 0.0184 | 0.0164 |
|  | Contribution of intra-region（%） | 53.85 | 52.07 | 53.10 | 55.36 | 54.57 | 51.32 | 54.95 | 3.39 | 47.15 | 37.84 | 31.95 |
|  | Contribution of inter-region（%） | 46.15 | 47.93 | 46.90 | 44.64 | 45.43 | 48.68 | 45.05 | 96.61 | 52.85 | 62.16 | 68.05 |
| **Screening of genetic metabolic disease** | intra-region | 1.030 | 0.468 | 0.309 | 0.154 | 0.116 | 0.0362 | 0.0385 | 0.0166 | 0.0035 | 0.0015 | 0.0007 |
|  | inter-region | 0.490 | 0.372 | 0.012 | 0.116 | 0.0717 | 0.0696 | 0.0435 | 0.5012 | 0.0426 | 0.0035 | 0.0018 |
|  | Contribution of intra-region（%） | 67.76 | 55.71 | 96.26 | 57.04 | 61.80 | 34.22 | 46.95 | 3.21 | 7.59 | 30.00 | 28.00 |
|  | Contribution of inter-region（%） | 32.24 | 44.29 | 3.74 | 42.96 | 38.20 | 65.78 | 53.05 | 96.79 | 92.41 | 70.00 | 72.00 |
| **Screening of hearing** | intra-region | 0.840 | 0.647 | 0.133 | 0.094 | 0.036 | 0.0225 | 0.0128 | 0.0059 | 0.0021 | 0.0013 | 0.0014 |
|  | inter-region | 0.430 | 0.347 | 0.014 | 0.143 | 0.1298 | 0.1028 | 0.0652 | 0.5236 | 0.037 | 0.0047 | 0.0024 |
|  | Contribution of intra-region（%） | 66.14 | 65.09 | 90.48 | 39.66 | 21.71 | 17.96 | 16.41 | 1.11 | 5.37 | 21.67 | 36.84 |
|  | Contribution of inter-region（%） | 33.86 | 34.91 | 9.52 | 60.34 | 78.29 | 82.04 | 83.59 | 98.89 | 94.63 | 78.33 | 63.16 |
| **Health management of children under 3** | intra-region | 0.070 | 0.069 | 0.07 | 0.077 | 0.0683 | 0.0669 | 0.0643 | 0.0143 | 0.0398 | 0.0158 | 0.1262 |
|  | inter-region | 0.040 | 0.038 | 0.003 | 0.05 | 0.0385 | 0.0435 | 0.0447 | 0.3001 | 0.0308 | 0.0145 | 0.0129 |
|  | Contribution of intra-region（%） | 63.64 | 64.49 | 95.89 | 60.63 | 63.95 | 60.60 | 58.99 | 4.55 | 56.37 | 52.15 | 90.73 |
|  | Contribution of inter-region（%） | 36.36 | 35.51 | 4.11 | 39.37 | 36.05 | 39.40 | 41.01 | 95.45 | 43.63 | 47.85 | 9.27 |

**Supplemental Table 2 Inequity of prenatal care utilization in four regions 2009-2019**

|  | **Reproductive health tests for brides-to-be** | | | | **Antenatal care**  **(at least five times)** | | | | **Health management of the pregnant women** | | | | **Psychological assessments and consultations** | | | | **Education classes for mother-to-be** | | | |
| --- | --- | --- | --- | --- | --- | --- | --- | --- | --- | --- | --- | --- | --- | --- | --- | --- | --- | --- | --- | --- |
|  | **PRD** | **N** | **E** | **W** | **PRD** | **N** | **E** | **W** | **PRD** | **N** | **E** | **W** | **PRD** | **N** | **E** | **W** | **PRD** | **N** | **E** | **W** |
| **2009** | 0.260 | 1.410 | 0.730 | 0.430 |  |  |  |  | 0.170 | 0.005 | 0.004 | 0.005 |  |  |  |  |  |  |  |  |
| **2010** | 0.220 | 0.570 | 2.104 | 0.208 |  |  |  |  | 0.130 | 0.005 | 0.002 | 0.001 |  |  |  |  |  |  |  |  |
| **2011** | 0.309 | 0.704 | 0.596 | 0.1175 | 0.1230 | 0.0050 | 0.0021 | 0.0024 | 0.1420 | 0.0045 | 0.0022 | 0.0024 |  |  |  |  |  |  |  |  |
| **2012** | 0.2446 | 0.5784 | 0.294 | 2.8894 | 0.1128 | 0.0019 | 0.0106 | 0.0397 | 0.1332 | 0.0017 | 0.0107 | 0.0385 |  |  |  |  |  |  |  |  |
| **2013** | 0.3468 | 0.696 | 0.0499 | 2.4675 | 0.1147 | 0.0028 | 0.0041 | 0.0013 | 0.1289 | 0.0026 | 0.0038 | 0.0013 |  |  |  |  |  |  |  |  |
| **2014** | 0.51 | 0.7099 | 0.0926 | 2.3689 | 0.0996 | 0.0036 | 0.0024 | 0.0015 | 0.1114 | 0.0038 | 0.0025 | 0.0018 |  |  |  |  |  |  |  |  |
| **2015** | 0.4936 | 0.8502 | 0.5441 | 1.1888 | 0.0915 | 0.0039 | 0.0026 | 0.0019 | 0.0960 | 0.0042 | 0.0021 | 0.0022 |  |  |  |  |  |  |  |  |
| **2016** | 0.2575 | 0.7949 | 0.4511 | 2.186 | 0.0685 | 0.0042 | 0.0025 | 0.0025 | 0.0709 | 0.0047 | 0.0027 | 0.0023 | 0.731 | 3.1228 | 0.0728 | 1.2165 | 0.0082 | 0.0512 | 0.0567 | 0.1733 |
| **2017** | 0.2735 | 0.2972 | 1.4817 | 0.0439 | 0.0375 | 0.0060 | 0.0013 | 0.0017 | 0.0416 | 0.0062 | 0.0016 | 0.0016 | 0.6109 | 3.4825 | 0.0808 | 1.1615 | 0.0064 | 0.0375 | 0.0567 | 0.1188 |
| **2018** | 0.2453 | 0.965 | 1.2767 | 0.4666 | 0.0189 | 0.0011 | 0.0003 | 0.0016 | 0.0209 | 0.0011 | 0.0004 | 0.0014 | 0.8466 | 3.3102 | 0.0447 | 1.0831 | 0.0078 | 0.03 | 0.0368 | 0.118 |
| **2019** | 0.2482 | 0.6209 | 0.4832 | 0.7192 | 0.0149 | 0.0024 | 0.0004 | 0.0008 | 0.0154 | 0.0031 | 0.0005 | 0.0005 | 0.2186 | 3.5063 | 0.0976 | 0.1464 | 0.005 | 0.0328 | 0.0028 | 0.0915 |

Note: PRD: Pearl River Delta; N: northern region; E: eastern region; W: western region.

**Supplemental Table 3 Inequity of perinatal and postnatal care utilization in four regions 2009-2019**

|  | **Postnatal visit for the mothers** | | | | **Postnatal visit for the babies** | | | | **Screening of genetic metabolic disease** | | | | **Screening of hearing** | | | | **Health management of children under 3** | | | |
| --- | --- | --- | --- | --- | --- | --- | --- | --- | --- | --- | --- | --- | --- | --- | --- | --- | --- | --- | --- | --- |
|  | **PRD** | **N** | **E** | **W** | **PRD** | **N** | **E** | **W** | **PRD** | **N** | **E** | **W** | **PRD** | **N** | **E** | **W** | **PRD** | **N** | **E** | **W** |
| **2009** | 0.140 | 0.005 | 0.004 | 0.007 | 0.140 | 0.005 | 0.004 | 0.010 | 0.030 | 1.610 | 4.603 | 0.061 | 0.260 | 0.190 | 3.701 | 0.461 | **0.130** | **0.004** | **0.013** | **0.006** |
| **2010** | 0.120 | 0.007 | 0.002 | 0.002 | 0.120 | 0.007 | 0.003 | 0.002 | 0.020 | 0.165 | 2.630 | 0.032 | 0.240 | 0.248 | 2.640 | 0.337 | **0.1300** | **0.0030** | **0.0130** | **0.0006** |
| **2011** | 0.1130 | 0.0054 | 0.0015 | 0.0032 | 0.1120 | 0.0056 | 0.0013 | 0.0034 | 0.0120 | 0.0420 | 1.8480 | 0.0160 | 0.1120 | 0.1890 | 0.2600 | 0.0285 | **0.1280** | **0.0030** | **0.0157** | **0.0017** |
| **2012** | 0.1033 | 0.0025 | 0.0018 | 0.0391 | 0.1017 | 0.0022 | 0.0049 | 0.0420 | 0.0037 | 0.0102 | 0.9023 | 0.0368 | 0.0641 | 0.0278 | 0.3005 | 0.0491 | **0.1274** | **0.0019** | **0.0148** | **0.0413** |
| **2013** | 0.1043 | 0.0036 | 0.0037 | 0.0022 | 0.0999 | 0.0034 | 0.0042 | 0.0015 | 0.0019 | 0.0045 | 0.7138 | 0.0006 | 0.0223 | 0.0022 | 0.1416 | 0.0077 | **0.1238** | **0.0032** | **0.0184** | **0.0003** |
| **2014** | 0.0933 | 0.0035 | 0.0018 | 0.0017 | 0.0921 | 0.0035 | 0.0044 | 0.0018 | 0.0015 | 0.0090 | 0.2114 | 0.0007 | 0.0110 | 0.0060 | 0.0941 | 0.0041 | **0.1171** | **0.0012** | **0.0244** | **0.0037** |
| **2015** | 0.0874 | 0.0038 | 0.0014 | 0.0021 | 0.0874 | 0.0037 | 0.0021 | 0.0020 | 0.0111 | 0.0049 | 0.2011 | 0.0001 | 0.0043 | 0.0038 | 0.0543 | 0.0083 | **0.1147** | **0.0033** | **0.0086** | **0.0000** |
| **2016** | 0.0660 | 0.0040 | 0.0024 | 0.0023 | 0.0646 | 0.0037 | 0.0015 | 0.0021 | 0.0020 | 0.0009 | 0.1015 | 0.0001 | 0.0012 | 0.0044 | 0.0283 | 0.0011 | **0.0928** | **0.0080** | **0.0005** | **0.0000** |
| **2017** | 0.0355 | 0.0055 | 0.0010 | 0.0015 | 0.0344 | 0.0047 | 0.0008 | 0.0017 | 0.0001 | 0.0003 | 0.0240 | 0.0002 | 0.0006 | 0.0010 | 0.0105 | 0.0010 | **0.0672** | **0.0089** | **0.0004** | **0.0013** |
| **2018** | 0.0184 | 0.0012 | 0.0004 | 0.0013 | 0.0185 | 0.0014 | 0.0016 | 0.0013 | 0.0000 | 0.0001 | 0.0101 | 0.0001 | 0.0002 | 0.0008 | 0.0073 | 0.0001 | **0.0248** | **0.0054** | **0.0036** | **0.0025** |
| **2019** | 0.0136 | 0.0025 | 0.0003 | 0.0004 | 0.0126 | 0.0023 | 0.0007 | 0.0004 | 0.0000 | 0.0000 | 0.0046 | 0.0000 | 0.0000 | 0.0008 | 0.0085 | 0.0001 | **0.2180** | **0.0025** | **0.0016** | **0.0006** |

Note: PRD: Pearl River Delta; N: northern region; E: eastern region; W: western region.
